# Changes in medicine prescription following a medication review in older high-risk patients with polypharmacy

**DOI:** 10.1007/s11096-018-0602-3

**Published:** 2018-02-17

**Authors:** Marian Z. M. Hurmuz, Sarah I. M. Janus, Jeannette G. van Manen

**Affiliations:** Dept. Health Technology & Services Research Faculty of Behavioural, Management & Social Sciences, Universty of Twente, P.O. box 217, 7500 AE Enschede, Netherlands

**Keywords:** Drug prescription, Elderly, Medication review, Polypharmacy, The Netherlands

## Abstract

*Background* The more (inappropriate) drugs a patient uses, the higher the risk of drug related problems. To reduce these risks, medication reviews can be performed. *Objective* To report changes in the prescribed number of (potentially inappropriate) drugs before and after performing a medication review in high-risk polypharmacy patients. A secondary objective was to study reasons for continuing potentially inappropriate drugs (PIDs). *Setting* Dutch community pharmacy and general medical practice. *Methods* A retrospective longitudinal intervention study with a pre-test/post-test design and follow-up of 1 week and 3 months was performed. The study population consisted of 126 patients with polypharmacy and with additional risk for drug related problems that underwent a medication review in five community pharmacies. The medication review was performed by the pharmacist in close cooperation with the general practitioner of each corresponding patient. *Main outcome measure* Number of (potentially inappropriate) drugs, and appropriateness of prescribed medicines. *Results* The average number of drugs a patient used 1 day before the review was 8.7 (SD = 2.9), which decreased (*p* < 0.05) to 8.3 (SD = 2.7) 1 week after the review, and to 8.4 (SD = 2.6) 3 months after the review. The average number of PIDs was initially 0.6 (SD = 0.8) per patient and decreased to 0.4 (SD = 0.6, *p* < 0.05). Twenty-two of the 241 initial drug changes (9%) were deprescribed during follow-up. Registered reasons for continuing PIDs are clinical or patients’ preferences. *Conclusions* Performing medication reviews in polypharmacy patients seems useful to continue at least in high-risk patients in The Netherlands. The time-consuming reviews could be limited to patients who are willing to change their medication.

## Impact of practice

- Current Dutch practice regarding medication reviews by a pharmacist and in cooperation with the general practitioner, should be continued and further disseminated.
- Before a medication review, the patients’ wishes should be assessed by the general practitioner and taken into account.

## Introduction

With aging, the risk of developing chronic diseases increases and often lead to the use of multiple drugs [1], referred to as polypharmacy [2]. The prevalence of polypharmacy ranges from 34 to 65% in older patients in different countries [1, 3–5]. As polypharmacy increases, so does the use of potentially inappropriate drugs (PIDs) which leads to a higher risk of drug related problems (DRPs) [1, 6–11]. Polypharmacy is also an important risk factor for preventable hospital admissions [7, 12–15]. The costs associated with avoidable admissions are estimated, for the Netherlands, at over 85 million euros [12]. It is predicted that the number of elderly with multi-morbidity will continue to increase, which makes polypharmacy an expanding societal issue [5].

Medication reviews aim to reduce preventable admissions and related costs. In the Netherlands, the Systematic Tool to Reduce Inappropriate Prescribing (STRIP) method [2] is used in community pharmacies to systematically assess the medication usage of an individual patient through a review by a physician, pharmacist, and the patient (and/or informal caregiver or other caretakers).

Several studies have been performed regarding the effectiveness of medication reviews. On the one hand, medication reviews show little or no effect in improving the quality of life, mortality, patients’ functioning, (re)admissions, and adverse drug events [16–21]. On the other hand, in some studies a decrease was found in the number of drugs per patient, DRPs, emergency department contacts, or inappropriate drugs [16, 17, 22–25].

As of now, there is limited explanation for these unexpectedly small or absent effects seen in previous studies. One explanation might be that only certain subgroups of patients might benefit from medication reviews, which does not become apparent when mainly focusing on older polypharmacy patients in general. This idea is supported by one study in high-risk patients [17] in which an effect was observed on the total number of prescribed medication. It is, however, not clear whether there is an effect on the prescription of inappropriate medicines as well, which could further explain the limited health gains –like hospital admissions or quality of life- that were also observed. In order to draw any firm conclusions on the usefulness of medication reviews, there is a need for studies that aim to get a closer look on individual effects and on effects in high-risk groups—with e.g. impaired renal function, or poor compliance. Also, additional insight into the stability of changes in medication and the possible continuation of PIDs after a review may be required to further understand the impact of medication reviews.

## Aim of the study

The aim of our study was to assess the changes in number and appropriateness of drugs prescribed in high-risk polypharmacy patients directly after and 3 months following a medication review. A secondary goal of the study was to investigate the reasons for possible continuation of PIDs.

## Ethics approval

Retrospective patient data from the pharmacies’ databases were used, and no insight in the full electronic patient records was provided. The data that is used in this study is processed anonymously, so that the data could not be traced back to patients. In all five pharmacies a confidentiality agreement was signed, so that the data would not be shared with others. Approval for ethical standards of this study has been granted by the Ethics Committee of the Faculty of Behavioral, Management and Social Sciences of the University of Twente.

## Method

### Study design

A retrospective longitudinal intervention study with a pre-test/post-test design was carried out. The pre-test measurements were performed 1 day before the review, and the post-test measurements 1 week and 3 months after the review.

### Study population

The study population consisted of older high-risk polypharmacy patients registered in five community pharmacies in the Eastern part of the Netherlands who underwent a medication review between January 2015 and April 2016. The inclusion criteria used in this study match with the criteria described in the Dutch guideline on polypharmacy in elderly [2]:

Age 65 years and older, using five or more types of drugs, having one or more of the following risk factors:

- impaired renal function (eGFR < 50 ml/min/1,73 m2);
- impaired cognition (dementia or indications of memory disorders and other cognitive disorders);
- increased risk of falling (patient fell at least one time in the preceding 12 months);
- signals of poor compliance; or
- not living independently (living in care or nursing home).

A sample size calculation was performed, which showed that 156 patients are needed (α = 0.05; β = 0.20) to detect a mean difference in the primary outcome (number of drugs) of at least 0.28 with a standard deviation of 1.25 [22].

### Intervention: performing the medication reviews

The medication reviews were initiated by the pharmacist and further carried out in close cooperation with the corresponding general practitioners (GPs) in six steps that match with the Dutch guideline on polypharmacy [2]: (1) pharmacist selects patients, asks patients for permission, collects data, (2) pharmacist carries out pharmacotherapeutic anamnesis, (3) pharmacist identifies (potential) pharmacotherapeutic-related problems, (4) pharmacist and GP set up a pharmacotherapeutic treatment plan, (5) GP establishes the pharmacotherapeutic treatment plan and participation is tailored to the capabilities of the patient, (6) GP executes and monitors the intended interventions. The medication reviews have been performed since 2015 by the pharmacists and GPs. They did not receive a training in reviewing, but were offered the stepwise method as a guideline. The stepwise method is based on the START and STOPP criteria [26].

### Data collection

The medication passports and medication profiles (a timeline of which drugs are used during which period during 1 year) of each patient, and the pharmacist’s notes were used to derive, for each patient, information on: (1) type and dose of drug(s) 1 day before the review, 1 week after the review and 3 months after the review; (2) reasons for (not) stopping or (not) adding a particular drug. Reasons for (not) stopping a drug, while the guidelines indicate the reverse, were additionally identified by a short online questionnaire presented to six GPs and 4 pharmacists involved in the medication reviews.

The Anatomical Therapeutical Chemical (ATC) codes of the medications were used to have a uniform international naming. For each patient, drugs were identified that are potentially inappropriate given this patient’s medical condition(s) and/or other medicine(s) used by screening the medication passports and profiles (1st author). For this purpose, we used the Dutch translation [27] of the revised STOPP-criteria (Screening Tool of Older Peoples’ Prescriptions) that has been developed in Europe [26]. The Dutch version is modified e.g. by excluding drugs that are not registered in The Netherlands [27]. If the drug used was not considered inappropriate according to the STOPP criteria, we defined it as appropriate. To examine the (in)appropriateness of the medications, a couple of diagnostics were obtained from the pharmacies: blood pressure, renal function, potassium level, sodium level, non-protein bounded calcium, beats per minute, HAS-BLED score, and respiratory failure (ρO2- and ρCO2-level). Only if the pharmacies did not have this information, the GPs were asked. The diagnostics non-protein bounded calcium and respiratory failure were not included in this study, because these were not available. Therefore STOPP criteria C2 (use of antiplatelet agents, clopidogrel and other agents in this group, dipyridamole, vitamin K antagonists, direct oral anticoagulants when patient has increased risk of bleeding) and G4 (use of benzodiazepines when patient has acute or chronic respiratory insufficiency), and partly STOPP criterion B8 (use of thiazide diuretics when patient suffers of hypercalcaemia) were excluded from this study.

### Data analysis

Statistical analyses were performed using SPSS version 23. Paired samples t-tests (with 95% confidence intervals) were performed on the variables ‘number of drugs’ and ‘number of potentially inappropriate drugs’. Descriptive statistics were used to evaluate how many times a (inappropriate) type of drug was added, stopped or had a dose modification.

## Results

### Patient characteristics

One hundred and twenty-six patients were included in this study. After 3 months, 118 patients could still be included. Eight patients could not be included at 3 months, because some died before the 3 months had passed and for others follow-up time of 3 months was not reached yet. The population consisted of more women than men (58.7% (n = 74) vs. 41.3% (n = 52)) and the mean age of the population was 76 years old (see Table 1).

**Table 1:** Baseline characteristics of study population. N = 126

Patients included in study	
1 day before review (n)	126
1 week after review (n)	126
3 months after review (n)	118
Gender	
Women [n (%)]	74 (58.7)
Age at review [m (SD)]	76 (7.4)

### Changes in drug prescription following the review

One week after the medication review, the total number of drugs decreased from 1100 to 1048, and the mean number of drugs per patient decreased from 8.7 (SD = 2.9) to 8.3 (SD = 2.7) (Table 2). A decrease was also seen for the number of PIDs (from 70 to 45) and mean number of PIDs (from 0.6 [SD = 0.8] to 0.4 [SD = 0.6]). After 3 months, the numbers decreased further, except for the mean number of drugs per patient. All average decreases were statistically significant.

**Table 2:** Total number, mean number, and range of drugs and PIDs, and total and mean number of elderly with PIDs

	N	Total number of drugs	Mean number (SD) drugs per patient	Range number of drugs	Range in changes in number of drugs	Total number of PIDs^a^	Mean number (SD) PIDs^a^ per patient	Range number of PIDs^a^	Range in changes in PIDs^a^
1 day before review	126	1100	8.7 (2.9)	5–21	–	70	0.6 (0.8)	0–5	–
1 week after review	126	1048	8.3 (2.7)*	3–20	− 5 to + 4	45	0.4 (0.6)*	0–4	− 2 to + 1
3 months after review	118	985	8.4 (2.6)*	3–18	− 4 to + 4	42	0.4 (0.6)*	0–3	− 2 to + 1

^a^PIDs = potentially inappropriate drugs

*P < 0.05 (paired samples *t* test, reference 1 day before review)

Looking at the individual level instead of means, one can see that the difference in number of drugs between 1 day before review and 1 week after review decreased with a maximum of five drugs per individual patient, and increased with a maximum of four drugs per individual patient (Table 2). In case of the PIDs, both 1 week and 3 months after review, the maximum decrease was two drugs, and maximum increase of one drug on an individual level.

One week after the medication review, 241 changes were made (Table 3) in 126 patients. In 48.5% (n = 117) of the modifications a drug was stopped, in 27% (n = 65) of the modifications a drug is added, and the remaining 24.5% (n = 59) were dose modifications. After 3 months, 73 additional changes (51 new, 22 change back to initial drug/dose) were seen adding up to 314 changes in total in 118 patients. Of these 314 changes at 3 months after the review, 23 (45%) were stopped drugs, 37 (33%) were added drugs, and 13 (23%) were dose modifications.

**Table 3:** Drug modifications 1 week and 3 months after the medication review

	Total number of modifications	Number of specified modifications (%)	Mean number (SD) of modifications per patient	Range of modifications	Total number of patients	Number of patients with modification (%)
Modification of dose						
1 week since review	241	59 (24.5)	0.5 (0.7)	0–3	126	48 (38.1)
3 months—new	51	11 (3.5)	0.1 (0.3)	0–2	118	11 (9.3)
3 months—back to initial drug^a^	22	2 (0.6)	0.0 (0.1)	0–1	118	2 (1.7)
Drug added						
1 week since review	241	65 (27.0)	0.5 (0.8)	0–4	126	48 (38.1)
3 months—new	51	30 (9.6)	0.3 (0.8)	0–5	118	17 (14.4)
3 months—back to initial drug^a^	22	7 (2.2)	0.1 (0.3)	0–3	118	5 (4.2)
Drug stopped						
1 week since review	241	117 (48.5)	0.9 (1.2)	0–6	126	70 (55.6)
3 months—new	51	10 (3.2)	0.1 (0.3)	0–2	118	8 (6.8)
3 months—back to initial drug^a^	22	13 (4.1)	0.1 (0.4)	0–2	118	10 (8.5)

^a^The modification was a switch back to the drug/dose already prescribed before the review

A little more than half of the dose modifications were a reduction in dose (52.5% (n = 31) after 1 week, 53.8% (n = 7) after 3 months) (not in Table). All other dose modifications concerned a dose increase.

From Table 4 it can be seen that the drugs that were most frequently added, stopped, and of which the dose was modified are from the groups ‘alimentary tract and metabolism’ and ‘cardiovascular system’. Of the group ‘alimentary tract and metabolism’ vitamins and minerals were most often added (19% (n = 19)), and of the group ‘cardiovascular system’, diuretics (8% (n = 5)), agents acting on the renin-angiotensin system (8% (n = 5)) and lipid modifying agents (6% (n = 4)) were mostly added. The drugs that were stopped most often in the ‘cardiovascular system’ group concern the same drugs (6% (n = 7), 6% (n = 7), 8% (n = 9) respectively), and in the group ‘alimentary tract and metabolism’ these drugs were drugs for constipation (4% (n = 5)) and diabetes (5% (n = 6)). Another group in which drugs are relatively frequently stopped is the ‘nervous system’. Here, it mostly concerns analgesics (8% (n = 9)). The drugs which dose was most often modified were drugs used for diabetes (9% (n = 5)), minerals (9% (n = 5)), cardiac therapy (10% (n = 6)), and agents acting on the renin-angiotensin system (7% (n = 4)). Three months after the review, we saw the same pattern in most common drug groups.

**Table 4:** Type of changes in total number of drugs (N = 241) that were changed in 126 patients 1 week after review

Drug group [n (%)]	ATC codes	Added (n = 65)	Stopped (n = 117)	Dose modification (n = 59)
Alimentary tract and metabolism	A	30 (46.2)	21 (17.9)	18 (30.5)
Blood and blood forming organs	B	5 (7.7)	11 (9.4)	0 (0)
Cardiovascular system	C	14 (21.5)	34 (29.1)	20 (33.9)
Dermatologicals	D	0 (0)	2 (1.7)	0 (0)
Genito urinary system and sex hormones	G	3 (4.6)	8 (6.8)	0 (0)
Systemic hormonal preparations, excl. sex hormones and insulins	H	1 (1.5)	1 (0.9)	1 (1.7)
Anti-infectives for systemic use	J	0 (0)	1 (0.9)	1 (1.7)
Antineoplastic and immunomodulating agents	L	0 (0)	1 (0.9)	1 (1.7)
Musculo-skeletal system	M	0 (0)	10 (8.5)	2 (3.4)
Nervous system	N	7 (10.8)	19 (16.2)	5 (8.5)
Respiratory system	R	2 (3.1)	3 (2.6)	8 (13.6)
Sensory organs	S	3 (4.6)	6 (5.1)	3 (5.1)

Forty-four out of the 45 PIDs seen 1 week after the review (Table 2) already existed before the review. Table 5 lists the reasons for not stopping a PID. The notes of the pharmacists concern several clinical reasons for continuing the PID. Most often it is registered that negative health effects are to be expected by a change in drug or dose (7% (n = 3)). The GPs and pharmacists were also asked for reasons for continuing PIDs in general and this revealed that 89% (n = 8) of them mention the preference of the patient as a reason, while clinical reasons are less frequently mentioned.

**Table 5:** Registered and mentioned reasons for continuing PIDs after the medication review

Reasons registered in pharmacists’ notes concerning the PIDs that were continued	N = 44
The health status of patient is that bad that the negative consequences of the drug are taken for granted	3 (6.8)
Patient experiences negative effects of stopping the drug	3 (6.8)
The potassium level will be under surveillance by the GP, when the potassium level rises, the drug will be stopped	2 (4.5)
Patient does not experience any adverse events of this drug	1 (2.3)
The drug has been stopped partially, now it is being used on an as needed base	1 (2.3)
The drug has not been stopped directly, but the patient has a scheme to reduce the drug in steps	1 (2.3)
Indication to stop the drug does not apply in this case, because the patient only has a little superficially ulcer in the duodenum	1 (2.3)
Instead of stopping the drug, the dose is lowered	1 (2.3)
There is no better alternative	1 (2.3)

Reasons mentioned in questionnaire filled in by the five GPs and four pharmacists involved in the medication reviews	N = 9
The patient disagrees with the change	8 (88.9)
The GP thinks the patient does not want to change	3 (33.3)
The patient had previous experience with the change without good outcomes	3 (33.3)
The specialist disagrees with the change	2 (22.2)
Fragile health status of the patient is seen as contraindication	2 (22.2)
The GP disagrees with the change	1 (11.1)
Expected that the patient will become disoriented as a result of changes	1 (11.1)

## Discussion

This study aimed to assess changes in number and appropriateness of prescribed medication in older polypharmacy patients following a medication review in general practice. The overall findings suggest that medication reviews may be able to reduce the number of drugs and the number of potentially inappropriate drugs and that the changes remain fairly stable at least in the first 3 months. The drug groups ‘alimentary tract and metabolism’ and ‘cardiovascular system’ consist of the leading types of drugs that undergo changes due to the medication reviews. Overall, there were more patients that stopped one or more drugs than patients that were prescribed one or more additional drugs. Reasons for continuing PIDs are mainly the (expected) negative effects of changes and the patient’s preference.

Although there were more drugs stopped than added, which is confirmed by two previous Dutch studies [28, 29], the average decrease in number of drugs between before and after the review was rather small. Lenaghan et al. [17] and Jódar-Sánchez et al. [22] also showed an average reduction of less than one drug per patient, which matches the current result. There is a chance that the selected pharmacies and the corresponding general practitioners already pay much attention to the medication of the patients which results in a small difference between before and after the review. However, the ultimate goal of medication reviews is not (only) to reduce the number of drugs, but primarily to improve the medication lists when needed, so that the drug use will be safer, i.e. less PIDs are prescribed. We observed a significant decrease in PIDs from 0.6 per patient to 0.4 per patient, both 1 week and 3 months after the medication review. Although this is on average a small difference, it might be meaningful on an individual level. In some patients, the number of PIDs decreased by two. Currently, there is conflicting evidence for overall effects of PIDs on the occurrence of adverse drug reactions, hospital admissions and other clinical outcomes [18]. Nevertheless, there may be more prominent effects in certain subgroups and therefore certain individual patients might particularly benefit from deprescription of PIDs whereas others might not. Since it is unclear which subgroups would benefit most, more research is needed to study the effects of PIDs on a variety of outcomes in high risk groups, e.g. older patients who are also frail or have specific comorbidities.

The small decreases in mean number of drugs and number of PIDs can also be explained by the finding that in some cases drugs that had to be stopped according to the guideline were actually not stopped. At first sight, it seems as if the STOPP criteria is not used well. However, for some specific patients, we identified reasons why it is better to keep using a drug, and to ignore the guidelines. Chau et al. [29] confirm this phenomenon. The most frequent reasons are: the specialist rejected the intervention; the patient did not want to stop the drug; and sometimes it is decided to monitor the use of the drug by the patient and to intervene when really needed. Besides these reasons, we identified two additional reasons that occurred relatively frequent. First, the health status of patients prevents stopping the drug, so the negative consequences of continuing the drug are taken for granted. Possible explanations for this could be that the GP and pharmacist are scared to disrupt a fragile balance when the patient stops that drug. This reason for not stopping the drug could well be understood by the high-risk population that is being studied. Second, patients tried to stop a drug before and experienced negative effects of stopping the drug, so they keep using it.

An important finding in our study was that, after the medication list of the patient has been assessed, patients sometimes refuse to undergo any changes in medication. Since patients are not informed beforehand in the current procedure, this would imply that the time-consuming reviews can be improved by explaining the medication review to patients and ask them whether they are open minded for changes before a review by the pharmacist and GP is planned. As a result, pharmacists and GPs may need to review fewer patients.

Striking in our study were the types of drugs that were stopped most frequently. Most stopped drugs belonged to the groups ‘alimentary tract and metabolism’, ‘cardiovascular system’, and ‘nervous system’. The types of the most frequently stopped drugs in these groups were drugs for constipation, drugs used in diabetes, diuretics, agents acting on the renin-angiotensin system, lipid modifying agents, and analgesics. Looking at existing literature, Mudge et al. [23] concluded that diuretics and analgesics were the most common deprescribed drugs. However, they also concluded that antiepileptics and psychoanaleptics were common deprescribed drugs, which differs from the results in the current study. In our study there were more cardiovascular drugs that were stopped frequently, which was also found in a previous Dutch study [28]. Possible, well-known explanations for these differences are first that antiepileptics are typically being prescribed to patients with epilepsy. When this is the case, the drug will not be stopped easily. Second, when antiepileptics are being prescribed as painkillers, the chance of stopping this drug is higher, but this concerns a very small group in the Netherlands. Third, psychoanaleptics are not frequently being prescribed for in the Netherlands.

Finally, looking at the dose modifications after the medication review, it can be concluded that there were just a little bit more dose reductions than dose increases. Balen et al. [26] also showed an almost equal number between dose reductions and dose increases. In their study, 57.7% of the dose modifications were reductions, which confirms the result found in the present study. This could be seen as beneficial since dose reductions will reduce costs and may imply a lower intake of drugs for a patient.

This study has some limitations. First, the underlying limitation that comes with a pre-test/post-test study design is that there is no control group. A disadvantage of this is that it is not completely certain whether drug modifications are solely the result of the medication review or would have occurred anyway, especially after 3 months. Nevertheless, since the differences between 1 week and 3 months after the review are minor, we assume this probably does not have a strong influence on the results. Still, this paper shows what changes may occur after a medication review, but we need to be careful in drawing the definitive conclusion that the changes are the result of the review. More research is needed in this respect.

Second, the GPs’ and pharmacists’ notes that are used in this study for identifying reasons for not stopping PIDs during the medication review do not cover all possible reasons. These are the only known reasons, because they are registered. Besides this, the occurrence rate of all the registered reasons could also be higher than shown in the results. Still, spontaneously registered reasons give some insight into the most important reasons for not stopping a drug.

Although we do not know how the medication reviews were carried out in practice, it is likely that there was (some) variation between pharmacists and between GPs. The results of this study therefore show what changes may occur after a medication review is performed based on a stepwise method as a guidance rather than based on a prescriptive protocol. Given the variation between pharmacists or between GPs, it is also likely that there is room for improvement of the medication reviews by providing for example a training on how to perform the reviews.

Furthermore, according to the power calculation, 156 patients were needed to detect a minimal difference of 0.28 in the number of drugs, but only 126 patients could be included in this study. However, in the 126 patients that were studied, the differences in the number of drugs were larger and a significant difference was already observed. It therefore would not have made a large difference if 30 more patients were included. Still, smaller differences that were actually present could not have been detected in this population and we therefore need to be careful to generalize the (insignificant) results to the population of elderly polypharmacy patients as a whole.

Finally, there is a possible chance that the pharmacists and GPs did not review the medicines the way they are supposed to do according to the guidelines. This can lead to a less complete review, for example, they may have overlooked some PIDs. However, we do not expect that this would affect our results significantly.

## Conclusion

This study adds to the limited and contradictory international literature regarding the changes in drug usage following medication reviews by studying the number of drugs and PIDs in older patients at increased risk for drug related problems. Performing medication reviews in polypharmacy patients -at least in high-risk patients in The Netherlands- can be useful to optimize drug use, despite the workload they bring with them. The optimized drug prescriptions seem to remain fairly stable after the review. Inclusion in the review of only those patients who are willing to modify their drugs could save time of the pharmacists and GPs.
